# Development and Characterization of Quinoa Peptide Nanoparticles as Carriers for Bioactive Food Ingredient Encapsulation

**DOI:** 10.3390/foods15091589

**Published:** 2026-05-04

**Authors:** Zulong Jin, Longhuan Duan, Xinyue Wang, Hongdong Song

**Affiliations:** 1 School of Health Science and Engineering, University of Shanghai for Science and Technology, Shanghai 200093, China; zulong.jin@sciex.com (Z.J.); spinachh@foxmail.com (L.D.); wangxinyue20210905@163.com (X.W.); 2 National Grain Industry (Urban Grain and Oil Security), Technology Innovation Center, Shanghai 200093, China

**Keywords:** quinoa peptide, caffeic acid phenethyl ester, water solubility, storage stability, in vitro release

## Abstract

Bioactive food ingredients offer significant health benefits. However, their poor water solubility and storage stability often limit their efficacy and practical application. In this study, quinoa peptide nanoparticles (QPNPs) were fabricated by controlled enzymatic hydrolysis, with particle sizes below 100 nm. Their structural stability was primarily maintained through hydrophobic interactions and hydrogen bonding. Caffeic acid phenethyl ester (CAPE) was selected as a model compound to evaluate the encapsulation performance of QPNPs. The results demonstrated that the encapsulation of CAPE was mainly driven by hydrophobic interactions and hydrogen bonding. The CAPE-loaded QPNPs (CAPE-QPNPs) exhibited a uniform particle size (194.1 ± 1.2 nm), high encapsulation efficiency (77.2%), and loading capacity (3.9%), significantly improving the water solubility and storage stability of CAPE. Furthermore, the cumulative release of CAPE in simulated gastrointestinal fluid was only 34% after 4 h, indicating strong resistance to digestion, which may be attributed to the dense shell structure of the nanoparticles. Overall, these findings suggest that QPNPs are a promising delivery system for encapsulating bioactive food ingredients and enhancing their physicochemical stability.

## 1. Introduction

Bioactive food ingredients offer significant health benefits; however, many of them, such as phenolic compounds, carotenoids, and fat-soluble vitamins, suffer from low water solubility and poor chemical stability, which greatly restrict their efficacy and practical applications [1]. For instance, caffeic acid phenethyl ester (CAPE), an important bioactive component found in propolis, has been widely reported to possess potent anticancer properties by effectively and selectively inhibiting cancer cell proliferation [2]. With the growing interest in health-promoting foods, CAPE has been extracted from propolis or chemically synthesized and incorporated into functional foods and dietary supplements. However, the catechol structure of CAPE is highly susceptible to oxidation due to its strong reducibility, leading to poor chemical stability [3]. In addition, the presence of two hydrophobic benzene rings results in low water solubility, further limiting its practical application [4]. Therefore, it is essential to develop effective strategies to improve both the water solubility and stability of such bioactive compounds.

To address these challenges, structural modification and nanoencapsulation are commonly employed approaches. However, chemical modification is often complex, costly, and less suitable for food applications. In contrast, nanosystems based on biomacromolecules are biocompatible, exhibit low toxicity, and can enhance the stability and bioactivity of encapsulated compounds [5]. Consequently, nanoencapsulation has attracted increasing attention as an effective strategy to improve the solubility and stability of bioactive food ingredients. For example, Tambuwala et al. developed an albumin nanocarrier to encapsulate CAPE, significantly enhancing its anti-inflammatory potential and ability to effectively modulate inflammation-related biomolecular pathways [6]. Similarly, Wei et al. prepared casein nanomicelles with a particle size of approximately 210 nm and an encapsulation efficiency of 96%, where hydrophobic interactions between casein and CAPE effectively protected CAPE from degradation, leading to improved bioavailability [7]. Despite these advantages, protein-based nanoparticles still exhibit limitations such as poor solubility, storage instability, and relatively low encapsulation efficiency, which restrict their broader application [8].

Peptide-based nanoparticles formed through the self-assembly of proteolytic peptides represent a promising alternative for bioactive compound delivery. Compared to protein-based systems, peptide nanoparticles exhibit several advantages, including improved solubility, resistance to aggregation, and enhanced loading capacity, making them particularly suitable for encapsulating hydrophobic compounds [9]. For instance, Ji et al. designed amphiphilic peptide nanoparticles capable of efficiently encapsulating hydrophobic drugs, demonstrating potential in antitumor therapy [10]. Lan et al. fabricated rapeseed-derived peptide nanoparticles and successfully encapsulated β-carotene, significantly improving its water solubility and stability by optimizing encapsulation conditions [11].

Quinoa proteins, primarily composed of albumins and globulins, have a balanced amino acid profile and are rich in essential amino acids such as lysine and histidine, making them a high-quality plant protein source [12,13]. In this study, quinoa peptide nanoparticles (QPNPs) were prepared via controlled enzymatic hydrolysis and ultrasonication-assisted self-assembly. CAPE was successfully encapsulated within QPNPs, and the encapsulation efficiency, loading capacity, water solubility, stability, and in vitro release behavior were systematically evaluated.

## 2. Materials and Methods

### 2.1. Materials

Quinoa was obtained from Yihe Agricultural Products Sales Co., Ltd. (Fanzhi, China). Alcalase 2.4L from Bacillus subtilis, trypsin from bovine pancreas, and α-chymotrypsin type II from bovine pancreas were purchased from Sigma-Aldrich (Shanghai, China). Caffeic acid phenethyl ester (CAPE, purity ≥ 97%) was obtained from Aladdin (Shanghai, China). All other chemicals and solvents, including sodium hydroxide (NaOH), hydrochloric acid (HCl), n-hexane, phosphotungstic acid, urea (U), dithiothreitol (DTT), and sodium dodecyl sulfate (SDS), were of analytical grade and purchased from Sinopharm Chemical Reagent Co., Ltd. (Shanghai, China).

### 2.2. Extraction of Quinoa Proteins

Quinoa was ground into powder using a grain mill (HK-820, Xulang Machinery Equipment Co., Ltd., Guangzhou, China) and passed through a 60-mesh sieve. The quinoa flour was defatted with n-hexane (1:3, *w*/*v*) under stirring for 2 h. The defatted powder was dispersed in distilled water and stirred at room temperature for 10 min to extract albumins. After centrifugation (3000× *g*, 10 min), the pH of the supernatant was adjusted to 4.5 and allowed to stand at 4 °C for 30 min to precipitate proteins. The precipitate was collected by centrifugation (3000× *g*, 15 min), washed twice with deionized water, and then freeze-dried under vacuum to obtain quinoa proteins.

### 2.3. Hydrolysis of Quinoa Proteins

Quinoa proteins were hydrolyzed using α-chymotrypsin, trypsin, and Alcalase 2.4L to produce self-assembling peptides. Briefly, quinoa protein solutions (10 g/L) were incubated with α-chymotrypsin (enzyme-to-substrate ratio 1:50, *w*/*w*, 50 °C), trypsin (1:25, *w*/*w*, 37 °C), or Alcalase 2.4L (1:50, *w*/*w*, 50 °C), respectively. The initial pH was adjusted to 8.0 and maintained at this value by adding 0.5 M NaOH. The degree of hydrolysis (*DH*) was monitored by the pH-stat method, as described by Bao et al. [14]. The *DH* was measured according to the following equation:

(1) $$DH(\%)=\frac{h}{h_{tot}}\times 100=\frac{bN_{b}}{aM_{p}h_{tot}}\times 100$$

where *b* and *N_b_* refer to the amount of NaOH during the proteolysis of the substrate and its normality, respectively; a represents the average degree of dissociation of the a-NH_2_ groups in the protein substrate; *M_p_* is the mass (g) of the protein; and *h_tot_* is the total number of peptide bonds in the protein substrate (8.2 mmol/g quinoa proteins).

Controlled enzymatic hydrolysis was performed until the *DH* reached 5% (α-chymotrypsin and trypsin) or 10% (Alcalase). The enzymatic reaction was terminated by heating in boiling water for 5 min to inactivate the enzyme. The hydrolysates were then centrifuged at 800× *g* to collect the clear supernatant containing soluble quinoa peptides. The peptide solution was subsequently freeze-dried to obtain peptide powders, which were stored at −20 °C until further use.

### 2.4. Preparation of Quinoa Peptides Nanoparticles (QPNPs)

The resulting peptide powders were dissolved in deionized water at a concentration of 1 g/L. The solutions were thoroughly mixed, followed by ultrasonication (240 W and 20 kHz) for 10 min, and then allowed to spontaneously self-assembly into nanoparticles at 4 °C overnight. Under these conditions, the formation of nanoparticles is likely driven by non-covalent interactions among amphiphilic peptide molecules [15]. The obtained nanoparticles were designated as Chy-QPNPs, Try-QPNPs, and Alc-QPNPs, respectively.

### 2.5. Particle Size and Polydispersity Index (PDI) Analysis

The particle size and polydispersity index (PDI) were determined using a dynamic light scattering (DLS) instrument (NanoBrook 173Plus, Brookhaven Instruments, Holtsville, NY, USA). Samples were dispersed in deionized water at a concentration of 1 mg/mL and equilibrated at room temperature for 30 min. Subsequently, 2.5 mL of the sample was transferred into a quartz cuvette for measurement. The scattering angle and refractive index of water were set at 90° and 1.333, respectively.

### 2.6. Determination of Zeta Potential

The zeta potential was determined using a Malvern Zetasizer (Nano ZS, Malvern Instrument Ltd., Malvern, UK). Briefly, 800 μL of the sample solution was loaded into a U-shaped cuvette, and measurements were carried out at room temperature. Ultrapure water was used as the dispersant, and the measurement angle was set at 90°.

### 2.7. Transmission Electron Microscope (TEM) Analysis

The morphologies of QPNPs and CAPE-loaded QPNPs (CAPE-QPNPs) were observed using a transmission electron microscope (JEM-2100, JEOL Ltd., Tokyo, Japan) according to a previously reported method [16] with some modifications. Briefly, 10 μL of sample (1 mg/mL) was dropped onto a carbon-coated copper grid and allowed to stand for 2 min. Excess liquid was removed using filter paper, followed by negative staining with phosphotungstic acid (1%, *w*/*v*) for 2 min. The samples were air-dried and observed at an accelerating voltage of 200 kV.

### 2.8. Surface Tension Measurements

Surface tension was determined using the pendant drop method with a contact angle analyzer (VCA Optima, AST Products, Inc., Billerica, MA, USA). QPNPs at different concentrations (0.01–5000 μg/mL) were dispensed through a needle to form pendant droplets. Droplet images were captured, and surface tension values were calculated from the droplet shape using the instrument’s software. Protein solutions at the same concentrations were used as controls. The surface tension of pure water was 72.66 ± 0.21 mN/m. All measurements were performed in triplicate.

### 2.9. Fourier Transform Infrared (FT-IR) Analysis

FT-IR spectra were recorded using an FT-IR spectrometer (Nicolet iS50, Thermo Fisher Scientific, Waltham, MA, USA). Samples were scanned over the range of 400–4000 cm^−1^ with a resolution of 4 cm^−1^, and each spectrum was obtained by averaging 64 scans. The spectra were analyzed using Origin software version 2026 to estimate the secondary structure of the samples according to a previously reported method [17].

### 2.10. Analysis of Internal Forces

The intermolecular interactions stabilizing QPNPs were evaluated according to a previously reported method [18]. Briefly, QPNPs were dispersed in solutions containing sodium dodecyl sulfate (SDS), dithiothreitol (DTT), and urea, either individually or in combination. The final concentrations of SDS, DTT, and urea were 0.5% (*w*/*v*), 30 mM, and 6 M, respectively. Changes in particle size were measured using a dynamic light scattering instrument (NanoBrook 173Plus, Brookhaven Instruments, Holtsville, NY, USA) to assess the contribution of different intermolecular forces to nanoparticle stability.

### 2.11. Encapsulation of CAPE in QPNPs (CAPE-QPNPs)

CAPE was first dissolved in ethanol (20 g/L) and then added to quinoa peptide solution (4 g/L) at a volume ratio of 1:200, in which relatively high encapsulation efficiency and loading capacity were achieved. The mixture was subjected to ultrasonication using an ultrasonic homogenizer (JN92-IIDN, Xinzi Biotechnology Instrument Co., Ltd., Ningbo, China) at 240 W and 20 kHz for 5 min (pulse mode: 3 s on, 2 s off). The ultrasonication conditions were selected based on preliminary trials to achieve stable nanoparticle formation and were not systematically optimized. Under these conditions, ultrasonic cavitation may partially disrupt the preformed peptide assemblies, leading to increased molecular dispersion and exposure of hydrophobic regions. This facilitates the incorporation of CAPE into the hydrophobic core of QPNPs. After ultrasonication, the system is allowed to equilibrate, during which peptides can reassemble into nanoparticles through non-covalent interactions. The resulting dispersion was centrifuged at 1000× *g* for 2 min, and this step was repeated five times to remove unencapsulated CAPE. Due to its poor aqueous solubility, free CAPE tends to precipitate or form aggregates and is thus removed in the pellet during centrifugation, whereas the well-dispersed CAPE-QPNPs remain in the supernatant. The supernatant containing CAPE-QPNPs was then collected for further analysis. The amount of encapsulated CAPE was quantified using an HPLC system (Shimadzu LC-16, Kyoto, Japan) equipped with a C18 reverse-phase column (4.6 mm × 250 mm). The mobile phase consisted of 80% methanol with 0.1% formic acid. The flow rate was 1.0 mL/min, the detection wavelength was 323 nm, and the run time was 10 min. Quantification of CAPE was performed using an external standard calibration curve. Standard solutions of CAPE at known concentrations were prepared, and a calibration curve was constructed by plotting peak area versus concentration (y = 1.48 × 10^−5^x + 1.48, where y is CAPE concentration, μg/mL and x is peak area). Encapsulation efficiency (EE) and loading capacity (LC) were calculated using the following equations:

(2) $$EE\left(\%\right)=\frac{\;\;amount\;of\;encapsulated\;CAPE}{total\;amount\;of\;CAPE}\;\;\times 100\%$$

(3) $$LC\left(\%\right)=\frac{amount\;of\;encapsulated\;CAPE}{weight\;of\;quinoa\;peptides}\times 100\%$$

### 2.12. Stability Evaluation of CAPE-QPNPs

#### 2.12.1. Thermal Stability

Freshly prepared free CAPE and CAPE-QPNPs solutions were incubated in a water bath at 25, 40, 55, 70, and 85 °C for 30 min. After incubation, the samples were immediately cooled to room temperature in an ice bath. The remaining CAPE content was determined using the HPLC method described above.

#### 2.12.2. Photostability

Freshly prepared free CAPE and CAPE-QPNPs solutions were exposed to UV irradiation (30 W, 254 nm) for 0, 1, 2, 4, and 6 h. The distance between the UV lamp and the samples was fixed at 70 cm. After exposure, the remaining CAPE content was quantified using the HPLC method described above.

#### 2.12.3. Storage Stability

Freshly prepared free CAPE and CAPE-QPNPs solutions were stored at 4 °C and 25 °C for 28 days. At predetermined time points (0, 3, 7, 14, 21, and 28 days), aliquots were collected to determine particle size and CAPE content using the methods described above.

### 2.13. In Vitro Digestion of CAPE-QPNPs

The in vitro digestion of CAPE-QPNPs was conducted using a simulated gastrointestinal model consisting of simulated gastric fluid (SGF) and simulated intestinal fluid (SIF). SGF contained pepsin (2.0 mg/mL) at pH 2.0 in phosphate-buffered saline (PBS), while SIF contained pancreatin (2.0 mg/mL) at pH 7.4 in PBS. For the gastric digestion phase, freshly prepared CAPE-QPNPs were mixed with SGF and transferred into a dialysis bag (molecular weight cut-off: 10 kDa), followed by incubation at 37 °C for 2 h. After gastric digestion, the pH was adjusted to 7.4 to inactivate pepsin, and the system was subsequently incubated in SIF at 37 °C for an additional 2 h. During digestion, the dialysis bag was immersed in PBS and gently shaken to allow the release of CAPE into the external medium. During digestion, PBS outside the dialysis bag maintained sink conditions and allowed the released CAPE to diffuse through the membrane into the external medium for quantification. At predetermined time points (0, 0.5, 1, 1.5, 2, 2.5, 3.0, 3.5, and 4 h), aliquots of the release medium were collected for CAPE quantification using the HPLC method described above. The cumulative release of CAPE was calculated using the following equation:

(4) $$Cumulative\;CAPE\;release\left(\%\right)=\frac{V_{0}C_{n}\;+\;V_{d}\sum_{1}^{n-i}C_{i}}{m_{i}}\;\times 100\%$$

where *V_0_* is the volume of the release medium, *C_n_* (mg/mL) is the concentration of CAPE in the release medium, *V_d_* is the volume of the medium taken out, *C_i_* is the concentration of CAPE in the released medium obtained for the i time, and *m_i_* (mg) is the initial CAPE amount in QPNPs.

### 2.14. Statistical Analysis

All experiments were performed in at least three replicates, and the values are expressed as the mean ± standard deviation (SD). Comparisons between two independent groups were performed using two-tailed unpaired Student’s *t*-tests. Differences among three or more independent groups were analyzed by one-way analysis of variance (ANOVA) followed by Tukey’s multiple-comparison test. When *p* was <0.05, the difference was considered to be significant.

## 3. Results and Discussion

### 3.1. Characterization of Quinoa Peptide Nanoparticles (QPNPs)

#### 3.1.1. Size, Polydispersity Index (PDI), and Appearance

**Figure 1:**
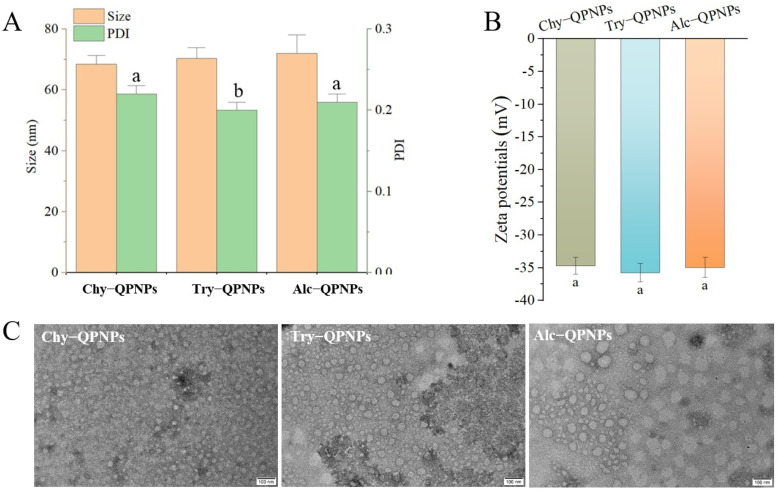
(**A**) Particle size and PDI of quinoa peptide nanoparticles (QPNPs). (**B**) Zeta potentials of QPNPs. (**C**) Transmission electron microscopy (TEM) images of QPNPs, scale bar: 100 nm. Chy-QPNPs, Try-QPNPs, and Alc-QPNPs refer to QPNPs prepared by controllable enzymolysis of α-chymotrypsin, trypsin, and Alcalase 2.4L, respectively. Different letters on columns mean significantly different (*p* < 0.05).

Figure 1A presents the particle size and polydispersity index (PDI) of peptide nanoparticles obtained via controlled enzymatic hydrolysis of quinoa proteins using α-chymotrypsin, trypsin, and Alcalase 2.4L. No significant differences in particle size were observed among the three types of nanoparticles, which may be attributed to their comparable degrees of hydrolysis. The PDI reflects the uniformity of particle size distribution, with lower values indicating narrower distributions. A PDI value below 0.5 is generally considered acceptable for nanoparticle systems [19]. In this study, the PDI values of the three QPNPs ranged from 0.20 to 0.22, indicating good dispersion and uniformity. Notably, Try-QPNPs exhibited a significantly lower PDI compared to Chy-QPNPs and Alc-QPNPs, suggesting a more homogeneous particle size distribution.

The zeta potentials of QPNPs were measured to further evaluate their colloidal stability. As shown in Figure 1B, all QPNPs exhibited negative surface charges of approximately −35 mV, with no significant differences among the groups (*p* > 0.05). These values indicate good colloidal stability, which is consistent with the observed dispersion behavior during storage (see below storage stability). The relatively high negative zeta potential may be attributed to the exposure of ionizable groups such as carboxyl groups on the peptide surface after enzymatic hydrolysis, contributing to electrostatic repulsion and stabilization of the nanoparticles.

Figure 1C shows the transmission electron microscopy (TEM) images of the three QPNPs. All samples exhibited spherical or near-spherical morphologies, indicating that quinoa protein-derived peptides were capable of self-assembling into well-defined nanostructures following enzymatic hydrolysis. The particle sizes observed by TEM were mainly in the range of 40–100 nm, which is consistent with the dynamic light scattering results shown in Figure 1A. Particle size plays a critical role in cellular uptake. Nanoparticles with sizes below 100 nm, particularly those around 50 nm, are more readily internalized by cells [20]. Therefore, the relatively small size of QPNPs suggests their potential for efficient uptake by intestinal epithelial cells following oral administration.

#### 3.1.2. Surface Tension, Secondary Structure, and Internal Forces

Surface hydrophilicity is a key property of peptide nanoparticles, as it directly influences their interaction with biological membranes and their ability to penetrate the intestinal barrier [21]. Nanoparticles with different hydrophilic–hydrophobic characteristics exhibit distinct behaviors in cellular uptake and transmembrane transport. In aqueous systems, amphiphilic or hydrophobic molecules tend to accumulate at the air–water interface, leading to a reduction in surface tension [22]. Therefore, surface tension can be used as an indirect indicator of the surface hydrophilicity of nanoparticles.

**Figure 2:**
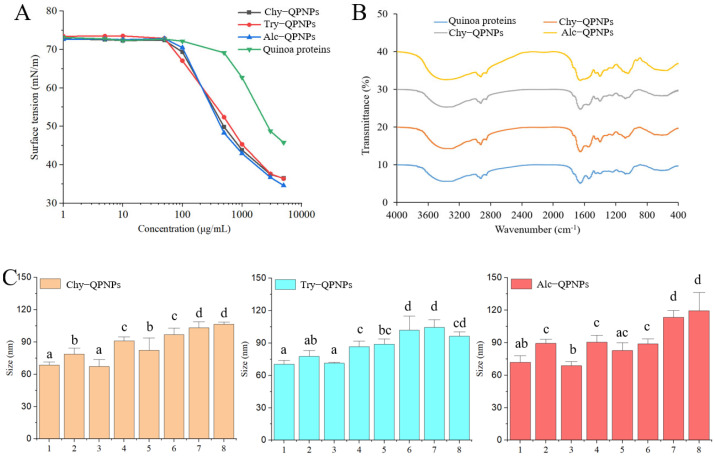
(**A**) Surface tension of quinoa peptide nanoparticles (QPNPs). (**B**) Fourier transform infrared spectroscopy (FT-IR) of QPNPs. (**C**) Effect of different denaturants on the particle size of QPNPs, 1: deionized water; 2: SDS; 3: DTT; 4: Urea; 5: SDS + DTT; 6: DTT + Urea; 7: SDS + Urea; 8: SDS + DTT +Urea. Different letters on columns mean significantly different (*p* < 0.05). Chy-QPNPs, Try-QPNPs, and Alc-QPNPs refer to QPNPs prepared by controllable enzymolysis of α-chymotrypsin, trypsin, and Alcalase 2.4L, respectively.

As shown in Figure 2A, the surface tension of peptide nanoparticle solutions remained relatively constant at concentrations below 90 μg/mL, but decreased significantly at higher concentrations. This phenomenon may be attributed to the adsorption of peptide nanoparticles at the air–water interface when the concentration exceeds a critical threshold, driven by exposed hydrophobic groups. In addition, at a concentration of 5 mg/mL, quinoa proteins exhibited a surface tension of approximately 45 mN/m, whereas peptide nanoparticle solutions showed lower values (35–37 mN/m). This decrease in surface tension can be attributed to enzymatic hydrolysis, which disrupts the native protein structure and exposes hydrophobic residues. These hydrophobic groups enhance surface activity and promote adsorption at the air–water interface, thereby reducing surface tension. This result is consistent with previous studies reporting that surface hydrophobicity increases with the degree of hydrolysis [23]. Among the three enzymes tested, Alcalase 2.4L produced peptide nanoparticles with the lowest surface tension, likely due to its broader cleavage specificity, which generates peptides with more exposed hydrophobic groups.

Figure 2B shows the Fourier transform infrared (FT-IR) spectra of quinoa proteins and peptide nanoparticles. The absorption band at 1700–1600 cm^−1^ corresponds to the amide I region, primarily associated with C=O stretching vibrations, while the band at 1550–1530 cm^−1^ represents the amide II region, mainly arising from N–H bending and C–N stretching vibrations. The broad band observed at 3500–3100 cm^−1^ is attributed to O–H stretching vibrations. Compared with quinoa proteins, the three QPNPs exhibited a red shift of the absorption peak around 3370 cm^−1^, indicating alterations in the hydrogen bonding environment, likely reflecting strengthened or rearranged intermolecular hydrogen bonding during peptide self-assembly. This may be attributed to the exposure of carboxyl groups generated during enzymatic hydrolysis, which participate in intermolecular hydrogen bonding [24]. In addition, a blue shift was observed in the amide II region (~1546 cm^−1^) for Try-QPNPs and Alc-QPNPs, which may be related to the disruption of original hydrogen bonding interactions, leading to shorter bond lengths and increased vibrational frequencies. These spectral changes suggest that enzymatic hydrolysis altered the secondary structure of quinoa proteins and promoted the formation of peptide-based nanostructures with distinct conformational features.

To further elucidate the intermolecular forces driving peptide self-assembly, SDS, DTT, and urea were used as denaturants, and the resulting changes in particle size were analyzed (Figure 2C). Treatment with DTT alone, a reducing agent that cleaves disulfide bonds, had no significant effect on particle size. However, a marked increase in particle size (in Try-QPNPs) was observed under combined treatments (DTT with urea), suggesting that disulfide bonds may contribute to structural integrity, particularly when other stabilizing forces are weakened. In contrast, both SDS and urea significantly increased the particle size of the nanoparticles. SDS disrupts hydrophobic interactions, while urea weakens hydrogen bonding [25]. The observed particle expansion suggests that both hydrophobic interactions and hydrogen bonds are essential for maintaining nanoparticle integrity. Notably, the combined treatment with SDS and urea resulted in the largest particle size, indicating a synergistic disruption effect. Overall, these results demonstrate that both hydrophobic interactions and hydrogen bonding contribute to the structural stability of QPNPs. This finding is consistent with previous reports on the intermolecular interactions governing soybean protein aggregates [26].

Table 1 summarizes the secondary structure composition of quinoa protein and peptide nanoparticles. The combined content of α-helix and β-sheet in quinoa protein, Chy-QPNPs, Try-QPNPs, and Alc-QPNPs was 64.1%, 64.5%, 61.6%, and 63.4%, respectively, indicating that enzymatic hydrolysis did not markedly alter the overall ordered structure content. Further analysis of the α-helix/β-sheet ratio revealed a significant increase in Chy-QPNPs compared with the native protein. This change may result from the disruption of β-sheet regions during enzymatic cleavage, followed by the formation of new hydrogen bonds that favor α-helix structures. An increase in β-sheet content is generally associated with enhanced structural rigidity and intermolecular interactions [27]. Such structural rearrangement suggests a decrease in the molecular rigidity of Chy-QPNPs. Meanwhile, enzymatic hydrolysis also led to an increase in β-turn content, indicating enhanced molecular flexibility. The coexistence of increased rigidity (α-helix) and flexibility (β-turn) suggests that peptide nanoparticles possess a balanced structural organization, which may be beneficial for nanoparticle stability and self-assembly. Given that the DH differed among enzymes (5% for α-chymotrypsin and trypsin vs. 10% for Alcalase), the observed differences cannot be attributed solely to enzyme specificity, but likely reflect the combined influence of enzyme type and hydrolysis extent. Considering that Try-QPNPs exhibited a smaller particle size and a narrower size distribution, they were selected for subsequent encapsulation of CAPE.

### 3.2. Characterization of CAPE-Loaded QPNPs (CAPE-QPNPs)

**Figure 3:**
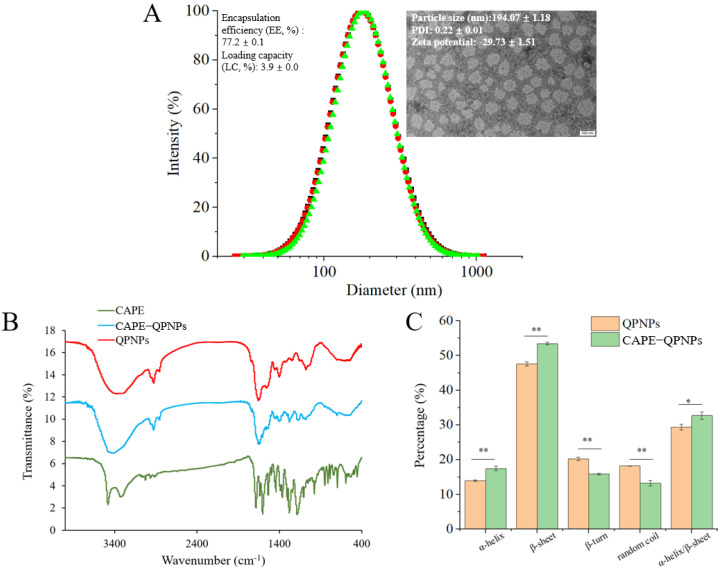
(**A**) Particle size distribution of CAPE-loaded QPNPs (CAPE-QPNPs) (Inset: TEM images of CAPE-QPNPs), the different colors represent three independent replicate experiments. (**B**) Fourier transform infrared spectroscopy (FT-IR) of CAPE, QPNPs, and CAPE-QPNPs. (**C**) Secondary structure proportion of QPNPs and CAPE-QPNPs, * *p* < 0.05, ** *p* < 0.01.

As shown in Figure 3A, the average particle size of CAPE-loaded QPNPs (CAPE-QPNPs) was approximately 194 nm, which was larger than that of blank QPNPs (72 nm), indicating successful encapsulation of CAPE within the nanoparticle structure. Transmission electron microscopy (TEM) images further confirmed the increase in particle size after encapsulation, consistent with the dynamic light scattering results. Ultrasound-assisted encapsulation has been widely reported as an effective strategy for preparing drug-loaded nanoparticles with minimal impact on material properties [28]. Following ultrasonic treatment, the apparent aqueous solubility of CAPE increased from 1.80 ± 0.11 μg/mL to 6.33 ± 0.37 μg/mL, suggesting that ultrasonication partially improved CAPE dispersion. Notably, after encapsulation into QPNPs, the apparent solubility of CAPE further increased to 154.50 ± 0.10 μg/mL, demonstrating the excellent solubilization capacity of peptide nanoparticles. The encapsulation efficiency (EE) and loading capacity (LC) of CAPE in QPNPs were 77.2% and 3.9%, respectively, indicating that QPNPs are effective delivery carriers for hydrophobic bioactive compounds. Although the encapsulation efficiency of QPNPs (77.2%) is lower than that reported for casein nanomicelles (~96%) [7], they present several distinct advantages compared to casein-based systems. First, as a plant-derived system, QPNPs align with the increasing demand for sustainable and vegan-friendly food ingredients. Second, QPNPs exhibited a smaller particle size and better performance in improving the water solubility and storage stability of CAPE. Third, the relatively low cumulative release (34% after 4 h, see the following part 3.4) suggests enhanced resistance to gastrointestinal digestion, which is beneficial for controlled release and prolonged bioactivity.

The FT-IR spectra of peptide nanoparticles before and after CAPE encapsulation are shown in Figure 3B. The characteristic peaks of CAPE were not clearly observed in the FTIR spectrum of CAPE-QPNPs, which is likely due to the relatively low loading amount of CAPE and the dominant contribution of the peptides. Therefore, FTIR alone cannot conclusively confirm the encapsulation of CAPE but may suggest the presence of interactions between CAPE and QPNPs. The amide I band (~1654 cm^−1^), corresponding to C=O stretching, showed no significant shift, while a slight blue shift was observed in the amide II band (~1546 cm^−1^), from 1535 to 1553 cm^−1^. This shift may be attributed to partial disruption of hydrogen bonding involving N–H groups, resulting in shorter bond lengths and increased vibrational frequencies. The minimal changes in amide band positions suggest that CAPE does not directly interact with peptide backbone amide bonds, and that non-covalent interactions-such as hydrophobic interactions and hydrogen bonding—likely dominate the encapsulation process.

As shown in Figure 3C, the secondary structure of QPNPs changed after CAPE encapsulation. Specifically, the contents of α-helix and β-sheet increased, while β-turn and random coil decreased. This indicates that the peptide nanoparticles adopted a more ordered structure upon encapsulation. This observation differs from previous reports in protein-based systems, where CAPE encapsulation did not significantly alter secondary structure [29]. The discrepancy may be attributed to the higher conformational flexibility of peptide-based systems compared to intact proteins, making them more susceptible to structural rearrangement upon interaction with hydrophobic compounds. The increased structural order is also consistent with the TEM observations, suggesting that CAPE encapsulation promotes a more compact and organized nanoparticle structure.

### 3.3. Stability of CAPE-QPNPs

**Figure 4:**
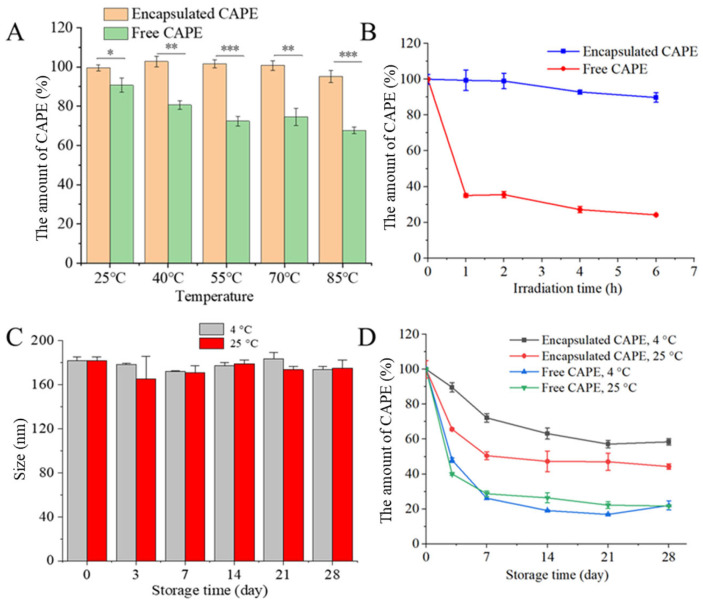
(**A**) Effect of temperatures on the remaining amount of CAPE in CAPE-loaded QPNPs (CAPE-QPNPs), * *p* < 0.05, ** *p* < 0.01, *** *p* < 0.001. (**B**) Effect of irradiation on the remaining amount of CAPE in CAPE-QPNPs. (**C**) Effect of storage time on the particle size of CAPE-QPNPs at 4 °C and 25 °C. (**D**) Effect of storage time on the remaining amount of CAPE in CAPE-QPNPs at 4 °C and 25 °C.

Stability is a critical factor for the practical application of CAPE-QPNPs. The thermal stability of CAPE-QPNPs was first evaluated (Figure 4A). The amount of CAPE (%) was calculated relative to the initial CAPE content (defined as 100%) at the beginning of each experiment. After heating at different temperatures for 30 min, the retention of CAPE in QPNPs remained above 90% across all conditions, showing minimal variation. In contrast, free CAPE exhibited significantly lower stability. These results indicate that peptide nanoparticles effectively enhance the thermal stability of CAPE.

CAPE is known to be sensitive to ultraviolet (UV) irradiation due to the presence of aromatic benzene rings, which readily absorb UV energy and undergo electronic transitions, leading to structural degradation [30]. As shown in Figure 4B, the retention of CAPE in QPNPs remained above 90% after 6 h of UV exposure, whereas free CAPE decreased to approximately 24%. This improved photostability may be attributed to the presence of aromatic amino acids, such as phenylalanine, tryptophan, and tyrosine, within the peptide nanoparticles, which can absorb or shield UV radiation and thereby protect encapsulated CAPE from oxidative degradation [31].

The storage stability of CAPE-QPNPs was further investigated at 4 °C and 25 °C over 28 days. As shown in Figure 4C, no significant changes in particle size were observed under either condition, indicating good physical stability of the nanoparticle system. This stability may be related to high negative zeta potential, strong hydrophobic interactions, and the compact structure of the peptide nanoparticles. Figure 4D shows the amount of CAPE during storage. The amount of CAPE at 4 °C was consistently higher than that at 25 °C throughout the 28-day period, suggesting that lower temperatures are more favorable for maintaining CAPE stability. Moreover, compared with free CAPE, encapsulated CAPE exhibited a significantly higher amount under both storage conditions, demonstrating that QPNPs effectively improve the storage stability of CAPE. The decrease in free CAPE under different conditions is likely due to its susceptibility to degradation (including thermal and photo-induced degradation), given its known instability in aqueous environments. In contrast, encapsulation within QPNPs provides a protective effect, thereby enhancing CAPE stability.

### 3.4. Release Behavior of CAPE-QPNPs in Simulated Gastrointestinal Digestion

**Figure 5:**
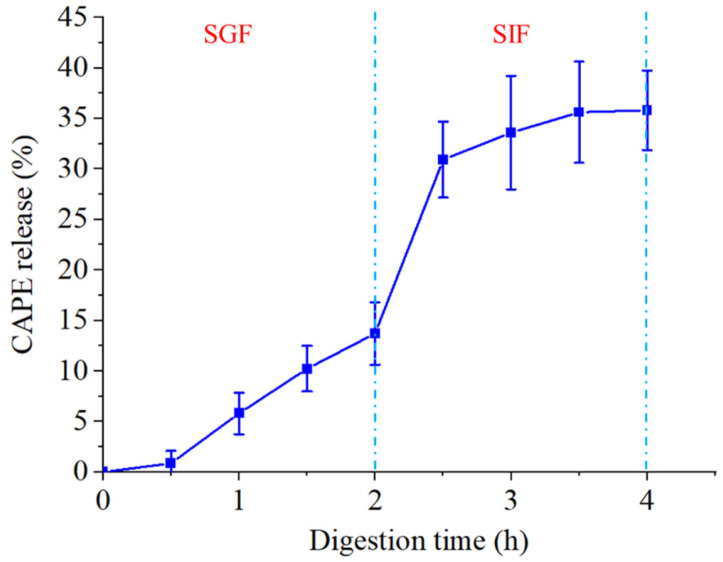
In vitro release profile of CAPE-loaded QPNPs (CAPE-QPNPs) in simulated gastric fluid (SGF) and intestinal fluid (SIF). The dotted lines indicate the transition from SGF to SIF and the end of the intestinal digestion phase, respectively.

In order to explore the digestion resistance of CAPE-QPNPs in gastrointestinal fluid, we performed an in vitro digestion simulation experiment, and the results are shown in Figure 5. The release profile of CAPE-QPNPs under simulated gastrointestinal conditions exhibited a clear stage-dependent behavior. During the simulated gastric phase (SGF, 0–2 h), only a limited amount of CAPE was released (approximately 14%), indicating that the nanoparticles remained relatively stable in acidic conditions. This stability may be attributed to strong hydrophobic interactions and hydrogen bonding within the nanoparticle structure. A sharp increase in CAPE release was observed upon transition to the simulated intestinal phase (SIF), with the release rate rising rapidly to approximately 30% at 2.5 h. This phenomenon is likely due to structural changes in the nanoparticles induced by pH variation and enzymatic digestion. Subsequently, the release rate gradually plateaued, reaching approximately 34% at 4 h, suggesting a sustained release behavior governed by diffusion and gradual nanoparticle disintegration. The relatively higher standard deviations observed during the SIF phase may be attributed to the more complex digestion environment, including enzymatic activity, pH changes, and structural rearrangement of the nanoparticles, which can result in increased variability in CAPE release. Although CAPE is much smaller than the molecular weight cut-off of the dialysis membrane (10 kDa) and can theoretically diffuse freely, the observed release was limited (~34% after 4 h). This suggests that the release of CAPE from QPNPs is not governed by membrane diffusion, but rather by its interactions with the nanoparticle matrix. Strong hydrophobic interactions and hydrogen bonding between CAPE and peptides may retain a significant fraction of CAPE within the nanoparticles, thereby slowing its release. In addition, the poor aqueous solubility of CAPE may further restrict its diffusion into the release medium. Therefore, the release behavior is likely governed by a combination of interaction-controlled release and solubility limitations. Compared with previously reported self-assembled sorghum peptide nanoparticles [25], QPNPs exhibited better performance in improving CAPE solubility, enhancing storage stability, and providing effective protection with a more sustained release behavior. Overall, these results demonstrate that QPNPs effectively protect CAPE under gastric conditions and enable controlled release in the intestinal environment, highlighting their potential as a delivery system for bioactive compounds.

## 4. Conclusions

In this study, QPNPs were successfully fabricated via controlled enzymatic hydrolysis. The obtained QPNPs exhibited particle sizes below 100 nm with a narrow PDI (0.20–0.22) and negative zeta potentials (−35 mV), indicating good uniformity and colloidal stability. Using CAPE as a model compound, QPNPs achieved an encapsulation efficiency of 77.2% and significantly improved the apparent solubility and storage stability of CAPE. Release studies showed that only ~34% of CAPE was released after 4 h under simulated gastrointestinal conditions, suggesting a sustained release profile. However, several limitations should be noted. A systematic optimization of formulation parameters was not performed. In addition, mass balance analysis and direct quantification of CAPE remaining in the dialysis system were not conducted, which limits a full understanding of the release mechanism. Future studies should focus on optimizing formulation parameters, performing comprehensive mass balance and stability analyses, and exploring the biological performance of QPNPs using in vitro and in vivo models. Overall, these findings demonstrate the feasibility of QPNPs as a plant-based nanocarrier for hydrophobic bioactive compounds and provide a basis for further development of peptide-derived delivery systems in functional food applications.

## Figures and Tables

**Table 1:** Secondary structure of quinoa peptide nanoparticles (QPNPs).

Samples	α-Helix (%)	β-Sheet (%)	β-Turn (%)	Random Coil (%)	α-Helix/β-Sheet (%)
Quinoa proteins	17.2 ± 1.7 ^b^	46.9 ± 1.8 ^a^	18.1 ± 0.4 ^a^	17.8 ± 0.5 ^a^	37.0 ± 5.1 ^b^
Chy-QPNPs	22.3 ± 0.4 ^a^	42.2 ± 0.0 ^b^	19.2 ± 0.2 ^b^	16.3 ± 0.6 ^b^	52.9 ± 1.0 ^a^
Try-QPNPs	14.0 ± 0.2 ^c^	47.6 ± 0.6 ^a^	20.2 ± 0.5 ^c^	18.2 ± 0.1 ^a^	29.4 ± 0.8 ^b^
Alc-QPNPs	16.0 ± 0.1 ^bc^	47.4 ± 0.8 ^a^	22.0 ± 0.3 ^d^	14.6 ± 0.6 ^c^	33.8 ± 0.4 ^b^

Note: Chy-QPNPs, Try-QPNPs, and Alc-QPNPs refer to QPNPs prepared by controllable enzymolysis of α-chymotrypsin, trypsin, and Alcalase 2.4L, respectively. Different letters mean significant difference (*p* < 0.05) between different samples in the same column.

## Data Availability

The original contributions presented in this study are included in the article. Further inquiries can be directed to the corresponding author.
